# Visual conservation treatment dilemmas in neuroblastoma with bilateral blindness

**DOI:** 10.1007/s12672-024-00898-1

**Published:** 2024-02-25

**Authors:** Haiyan Cheng, Yu Lin, Wei Yang, Xiaofeng Chang, Jun Feng, Shen Yang, Shan Liu, Tong Yu, Xiaojiao Peng, Panpan Zheng, Chengyue Zhang, Haiwei Jia, Hong Qin, Huanmin Wang

**Affiliations:** 1 Department of Oncology Surgery, Beijing Children’s Hospital, National Center for Children’s Health, Capital Medical University, Beijing, 100045 China; 2 Medical Imaging Center, Beijing Children’s Hospital, National Center for Children’s Health, Capital Medical University, Beijing, 100045 China; 3 Department of Neurosurgery, Beijing Children’s Hospital, National Center for Children’s Health, Capital Medical University, Beijing, 100045 China; 4 Department of Ophthalmology, Beijing Children’s Hospital, National Center for Children’s Health, Capital Medical University, Beijing, 100045 China; 5 Department of Radiotherapy, Beijing Fengtai You Anmen Hospital, Beijing, 10069 China; 6 MOE Key Laboratory of Major Diseases in Children, Beijing Children’s Hospital, National Center for Children’s Health, Capital Medical University, Beijing, 100045 China

**Keywords:** Children, Neuroblastoma, Bilateral blindness, Treatment

## Abstract

**Objective:**

To investigate the clinical features, treatment strategies, and prognosis of neuroblastoma with bilateral blindness.

**Methods:**

The clinical data of five patients with bilateral blindness neuroblastoma admitted to Beijing Children’s Hospital from April 2018 to September 2020 were retrospectively collected to summarize their clinical characteristics.

**Results:**

All patients were female and the median age at presentation was 25 (23, 41) months. The median intervention time from the onset of symptoms of bilateral blindness to the start of treatment was 10 (10, 12) days. All five cases were staged as stage M and grouped as high risk. Four cases were *MYCN* gene amplification and one case was *MYCN* acquisition. Five children were treated according to a high-risk neuroblastoma treatment protocol. Four children did not recover their vision after treatment, and one case improved to have light perception. All patients were effectively followed up for a median of 20 (12, 31) months, with three deaths, one tumor-free survival, and one recurrent tumor-bearing survival.

**Conclusion:**

Neuroblastoma with bilateral blindness is rare in the clinic, mostly in children of young age, and is often associated with *MYCN* amplification and multiple metastases. Early hormone shock therapy and optic nerve decompression are beneficial for preserving the child’s vision. A joint multi-disciplinary treatment may help in the formulation of treatment decisions. Achieving a balance between good visual preservation and survival within the short optic nerve neurotherapeutic window is extremely challenging.

## Background

Neuroblastoma (NB) is the most common extracranial solid tumor in children, accounting for approximately 8% of childhood cancers [1, 2]. It originates from the sympathetic nerve chain and is most commonly found in the abdomen. Most patients (> 90%) are diagnosed with NB before the age of 10 years; however, up to 50% have metastases at the time of diagnosis, usually in the bone marrow or bone. When the bone metastases compress the optic nerve or optic cross, it can lead to visual impairment, and the loss of binocular vision is one of the rare complications of the disease [3]. Currently, clinicians are not aware of this complication and the literature remains dominated by case reports, while the visual outcome and prognosis of such children are poor [4, 5]. Therefore, this study summarizes the clinical characteristics of five children with neuroblastoma combined with binocular blindness admitted to our center to discuss their clinical features, treatment, and prognosis to improve clinicians’ understanding of this complication and the possibility of saving the vision of patients such as these.

## Information and methods

The clinical data of 474 children with pelvic and abdominal neuroblastoma admitted to Beijing Children’s Hospital from April 2018 to September 2020 were retrospectively collected. Among them, 5 cases exhibited binocular blindness. The patients’ primary and metastatic tumor sites were identified through imaging (including PET-CT, MRI, enhanced CT, and ultrasound). Neuroblastoma was confirmed by postoperative pathology, and chemotherapy was preceded as an emergency and followed by tumor removal without histological diagnosis at the time of initial diagnosis. The INRG staging system was used to define the four stages: L1, L2, M, and Ms [6]. The INRG grouping was used to define very low-risk, low-risk, intermediate-risk, and high-risk groups [7]. The children’s survival was monitored through telephone follow-up and outpatient review records until 21 December 2022. The research was carried out following the guidelines of the ethics committee listed in the ethics statement. This retrospective study was approved by the Medical Ethics Committee of the Beijing Children’s Hospital (2017-k-89), and the patient informed consent requirements were waived by the Medical Ethics Committee of the Beijing Children’s Hospital.

Inclusion criteria: age < 18 years; clear postoperative pathology of neuroblastoma; clinical presentation of binocular blindness; orbital imaging evidence suggestive of the metastatic tumor compressing the optic nerve or optic cross.

Exclusion criteria: the presence of other possible causes of binocular blindness.

## Result

Five children with neuroblastoma with binocular blindness were admitted to our clinical center from April 2018 to September 2020. All were girls with a median age of 25 (22.5, 44.0) months. The tumors originated from the left adrenal gland in two cases, the right adrenal gland in two cases, and the right paravertebral in one case. The initial median tumor diameter was 7.8 (6.7, 12.6) cm. The initial clinical manifestations were that all patients had manifestations other than bilateral visual loss, including two cases with bilateral orbital bruising with ocular protrusion, one case with right orbital bruising, one case with left brow arch swelling, and one case with left ocular protrusion. The tumor markers including neuron-specific enolase (NSE), lactate dehydrogenase (LDH), vanilloid amygdalin/urinary creatinine, hyper vanilloid/urinary creatinine, and ferritin were measured and found to be significantly above the normal range in all five children in this group. In terms of the pathology of the primary focus, the postoperative pathological findings were returned as neuroblastoma. For the molecular biological features, there were four cases of *MYCN* gene amplification, one case of *MYCN* gene acquisition, two cases of 1p deletion, one case of 1p heterogeneous focal amplification, one case of 11q deletion, and one case of 1p imbalance with 11q acquisition (Table 1).

**Table 1:** Clinical characteristics of the five children

Case	Month age	Tumor size at first diagnosis (cm)	The primary site of the tumor	Metastatic site	NSE (ng/mL)	LDH (U/L)	VMA/Crn (%)	HVA/Crn (%)	VMA/HVA	Ferritin (ng/mL)	*MYCN*	1P	11Q
1	41	6.7	L adrenal	Bone/BM/liver	1350	3319	97.438	49.236	> 1	479.3	Amplification	Deletion	Normal
2	23	12.6	R adrenal	Bone/BM/pleura/distant LN	814.5	1324	353.419	25.004	> 1	–	Amplification	Normal	Deletion
3	48	14.8	R paravertebral	Bone/BM/distant LN	1410	4245	5.765	–	–	552.9	Amplification	Deletion	Normal
4	25	3.3	L adrenal	Bone/pleura/distant LN	> 370	2026	–	–	–	430.5	Amplification	Imbalance	Acquisition
5	22	7.8	R adrenal	Bone/BM	930	2398	136.266	15.545	> 1	110.1	Acquisition	Heterogeneous focal amplification	Normal

*BM* bone marrow, *LN* lymph node

All molecular biological results are from specimens obtained by total removal after chemotherapy

**Fig. 1:**
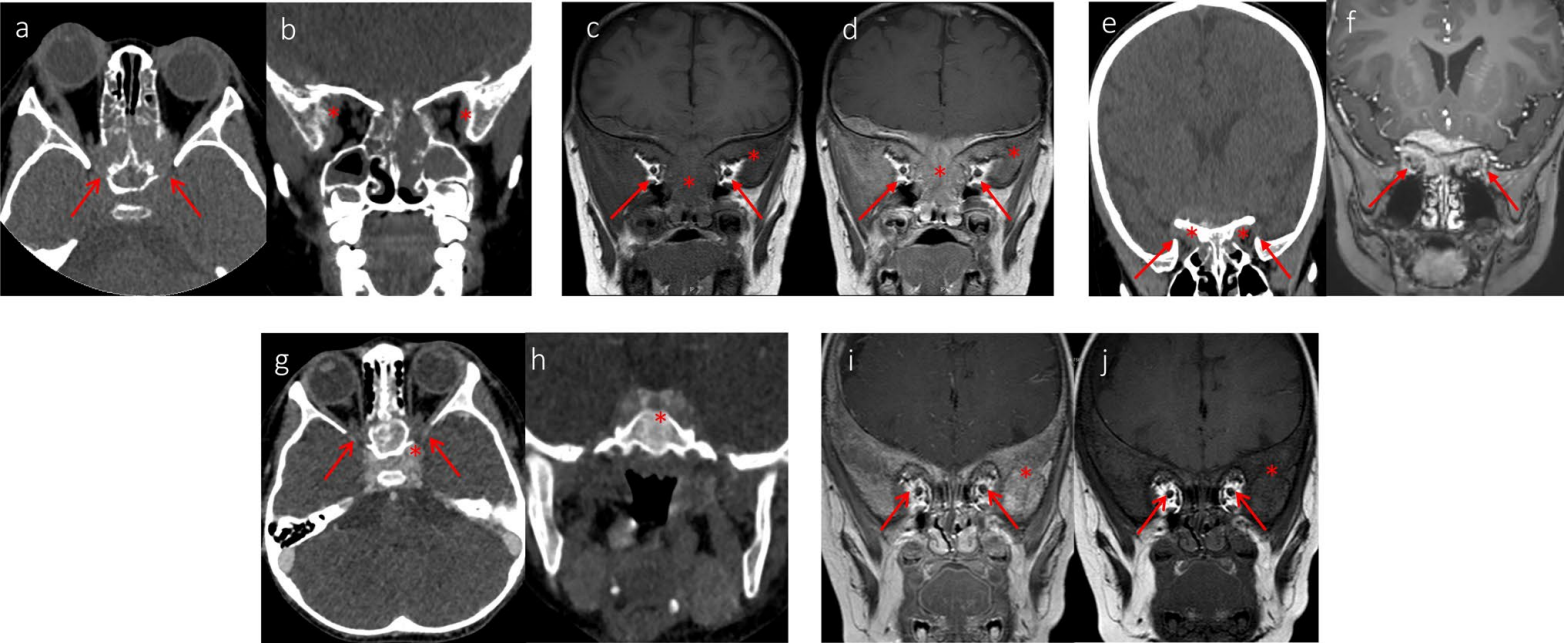
Head imaging of five children. **a**, **b** CT appearance of the head metastatic tumor in Case No. 1; **c**, **d** MRI appearance of the head metastatic tumor in Case No. 2; **e** CT appearance and **f** MRI appearance of the head metastatic tumor in Case No. 3; **g**, **h** CT appearance of the head metastatic tumor in Case No. 4; **i**, **j** MRI appearance of the head metastatic tumor in Case No. 5. Metastatic tumor lesion involving the optic foramen with compression of the optic nerve (arrow) and bone metastatic lesion with bone destruction and soft tissue formation (asterisk)

Ophthalmologic treatment and prognosis: Case No. 1 was admitted with “3 weeks of bilateral orbital petechiae and 2 weeks of proptosis”, and on the 4th day after admission, the patient developed blurred vision. Following an ophthalmologic consultation, bilateral para-optical radiation was still present, and periorbital enhanced CT and MRI were completed (Fig. 1). Meanwhile, the visually evoked potential (VEP) was improved, suggesting an atypical waveform, and the metastatic tumor was considered to involve the optic nerve. The patient progressed to bilateral blindness despite conservative treatment. The remaining four patients had bilateral blindness before admission and were not given ophthalmology-related treatment after admission except for oncology treatment.

In Case No. 4, after the completion of oncological treatment, regular ocular rehabilitation physiotherapy was performed for 1 year, and the visual acuity recovered from blindness to light perception and the ability to distinguish between day and night.

Oncological treatment and prognosis: five children were treated according to a high-risk neuroblastoma treatment protocol, in which the chemotherapy regimen alternated between the CAV regimen (cyclophosphamide, adriamycin, and vincristine) and the CVP regimen (cisplatin and etoposide) with the resection of the primary tumor, and postoperative maintenance chemotherapy, radiotherapy. Case No. 1 died due to chemotherapy complications with a survival period of 20 months. Case No. 2 died of distant tumor recurrence with a survival period of 18 months. Case No. 3 was found to have tumor recurrence 29 months after diagnosis and is currently surviving with a tumor with a survival period of 30 months.

Case No. 4 was diagnosed with recurrence 28 months after diagnosis, and was treated with bevacizumab and VI (vincristine and irinotecan) at the local oncology hospital. The child is currently living tumor-free, and the survival period is 32 months. Case No. 5 died after abandoning treatment due to bone marrow relapse, with a survival period of 6 months (Table 2).

**Table 2:** Treatment and survival of the five children

Case	Clinical manifestations other than blindness	Visual evoked potential	Number of days of vision loss found	Ophthalmology treatment options	Oncology treatment options	Recovery of vision	Outcome	Survival period (months)
1	Bilateral orbital bruising with oculars protrusion	Bilateral atypical waveforms	0	Mecobalamin + mannitol	Chemotherapy–surgery–chemotherapy	Not restored	Death	20
2	Right orbital bruising	Moderately reduced P2 wave amplitude bilaterally, with approximately normal latency	20	None	Chemotherapy–surgery–chemotherapy–radiotherapy	Not restored	Death	18
3	Swelling in the left arch of the eyebrow	–	10	None	Chemotherapy–surgery–chemotherapy–radiotherapy	Not restored	Survival with tumor	30
4	Left ocular protrusion	Mildly reduced P2 wave amplitude in the right with normal latency	10	Rehabilitation physiotherapy	Chemotherapy–surgery–chemotherapy–radiotherapy–immunotherapy and chemotherapy	Light-perception	Tumor-free survival	32
5	Bilateral orbital bilateral orbital bruising with oculars protrusion	Mildly reduced P2 wave amplitude in the right with normal latency	12	None	Chemotherapy–surgery–chemotherapy	Not restored	Death	6

## Discussion

NB is the most common extracranial solid tumor in children, accounting for 8% of childhood tumors. It originates from the adrenal gland or sympathetic chain and presents with signs and symptoms that depend on the anatomical site involved [8]. Among these ocular manifestations are Horner’s syndrome, pupillary inequality, iris heterochromia, ocular displacement, convergent strabismus, fixed and dilated pupils, optic nerve head atrophy, retinal edema, and optic clonus [9, 10]. However, binocular blindness is an extremely rare complication of neuroblastoma and is still mostly reported on a case-by-case basis.

The previous literature has reported a high proportion of children with blindness presenting with ocular-related manifestations such as protruding eyes, ocular swelling, and ocular petechiae [11]. In this study, according to the medical history of the children, five children had bilateral orbital bruising with proptosis, unilateral orbital bruising, and unilateral brow arch swelling in addition to bilateral visual loss. Irritable agitation and anorexia have also been reported in the previous literature [4, 12]. While binocular blindness manifested very early in the disease for the five patients, previous literature reports indicate that blindness can occur during tumor progression and recurrence [11]. We should be aware of visual impairment during treatment. Further, five children had significantly higher tumor-related parameters in laboratory tests with *MYCN* gene and chromosomal abnormalities and had developed systemic multiple metastases, and previous case reports also mentioned that *MYCN* amplification and systemic multiple metastases were found [9, 13]. Therefore, clinicians are warned that although binocular blindness is a rare complication associated with neuroblastoma, when associated periorbital clinical manifestations, significantly elevated tumor markers, multiple metastatic tumor foci throughout the body, or adverse molecular biological features are present, the child should be alerted to the possibility of blindness, and early detection and treatment should be sought to balance vision preservation and survival.

According to the imaging data of this group of children, “primary focal bone metastases to the optic canal or septal sinus, followed by the formation of metastases compressing the optic nerve or optic cross” were the cause of visual loss. Although the underlying pathophysiology of optic nerve compression-induced visual impairment is still being explored [14], some researchers believe that damage to retinal ganglion cells (RGC) caused by various factors further leads to a series of complex processes such as abnormal intracellular signaling pathways and the release of inflammatory factors such as interleukins and tumor necrosis factors, triggering an apoptotic program that ultimately leads to blindness [15].

For the child, their family, and their clinicians, permanent loss of vision in both eyes is a tremendous shock, meaning a severe loss of function and a significant reduction in the child’s quality of life [16]. Therefore, saving as much vision as possible remains a necessity for children with malignancy. In children with neuroblastoma with binocular blindness, treatment relies on close multi-disciplinary treatment (MDT). The related departments include Medical Oncology, Surgical Oncology, Radiotherapy Oncology, Ophthalmology, Neurosurgery, and Imaging. Potential treatments include hormonal shock therapy, induction chemotherapy, optic nerve decompression, and radiotherapy. The timing of treatment is still controversial, with some believing that hormonal treatment within 8 h of the onset of compression and surgical treatment within 12 to 24 h is the most effective approach [3, 4, 17, 18]. It has been suggested that the greatest benefit from optic nerve decompression surgery is achieved within 3 days of the onset of symptoms [19]. For oncology treatment, early induction chemotherapy can help control tumor progression. In any case, early intervention in treatment is beneficial [20]. The five children in this study had a median age of onset of 25 (22.5, 44.0) months and were all preschoolers. Belgaumi’s report referred to infants with neuroblastoma experiencing blindness [11]. Children of this age cannot express their subjective perceptions, so when the family notices that the child has a visual impairment, the optic nerve has often been involved for some considerable period of time. The median intervention time from the detection of visual impairment to clinical treatment in this group of patients was 10 (5, 16) days, while the actual time between optic nerve involvement and therapeutic intervention was greater than that. Therefore, the management of children with neuroblastoma with bilateral blindness should be considered an oncological emergency.

Early hormonal shock therapy and optic nerve decompression appear to be proven treatments for restoring vision in children with binocular blindness. One case of the reversal of blindness following high-dose hormonal shock and decompression surgery has been reported in the literature [18]. Hormone shock therapy is effective in cases where the optic nerve is compressed, squeezed, or stretched, resulting in impaired function, with approximately 50% showing significant improvement in vision after hormone therapy, which is routinely administered in high-dose hormone shock therapy [4, 18, 21, 22]. In addition, early induction chemotherapy can help control tumor progression in terms of the oncology treatment pathway [20], but chemotherapy itself is a blow and is often associated with complications such as bone marrow suppression and liver and kidney cardiotoxicity, making the child less tolerant of anesthesia and surgery and possibly delaying decompression surgery. Induction chemotherapy after optic nerve or optic cross decompression surgery before the wound has recovered may contribute to the development of infection and non-healing wounds, while delaying chemotherapy must be accompanied by the risk of progression of the primary tumor and metastases. Thus, within the short treatment window for the optic nerve, clinicians may be faced with a dilemma in making treatment decisions.

Are there other treatment options to consider besides surgical decompression with induction chemotherapy to reduce the tumor load? Radiotherapy may be another option to save vision. Firstly, radiotherapy is recommended for children with high-risk neuroblastoma [23]. At the end of consolidation chemotherapy, a “radiotherapy dose of 21.6 Gy to the tumor bed” was administered to achieve better local control. The retina tolerates 30 Gy of radiotherapy, while “post-radiotherapy peripheral nerve injury” occurs at doses up to 60 Gy in periorbital radiotherapy. Therefore, theoretically, local radiotherapy can be completed at a locally controlled dose to the residual lesion as tolerated by the optic nerve. It has been reported in previous literature that radiotherapy is effective in the local control of tumors [24]. Radiotherapy is also used in the treatment of intravertebral neuroblastoma to reduce the size of the tumor. Additionally, in the treatment of intradural neuroblastoma, radiotherapy is used to shrink the tumor foci while relieving tumor compression resulting in epidural spinal cord compression (ESCC) such as lower limb weakness and motion-related pain [25]. Previous studies have shown that radiotherapy is effective in treating primary tumor sites as well as bone and soft tissue metastases [26]. Therefore, in children with bilateral blindness, periorbital radiotherapy may be attempted to save vision, while early induction chemotherapy may be further attempted if the child’s general condition is acceptable, in the hope of achieving a balance between good visual outcomes and survival. Due to the limited intracranial volume and the bony structure of the optic nerve canal, the local edema of the tumor early in radiotherapy can induce orbital apical syndrome, which can aggravate the compression of the optic nerve, and the prophylactic application of hormones can reduce the occurrence of the modified complications. In addition to the early complications of radiotherapy, distant complications of radiotherapy such as radiographic cataracts should also be taken into account. Therefore, in children with bilateral blindness, the treatment should be multidisciplinary, individualized, and based on the wishes of the family, with an early search for the best treatment strategy between decompression surgery, induction chemotherapy, and periorbital radiotherapy.

In one child in this study, after 1 year of rehabilitation physiotherapy at the end of oncological treatment, the visual loss improved compared to the previous situation; the child recovered from vision loss to perceptible light, suggesting that rehabilitation physiotherapy may have a role in the recovery of visual acuity. Additional treatments such as nerve-nourishing drugs, improved microcirculation, and dehydration to lower cranial pressure appear to play an adjunctive role in the treatment process.

This is a retrospective study with a small sample and some clinical information is missing. Because of the rarity of this complication in clinical practice, clinicians are not aware of neuroblastoma with binocular blindness and therefore none of the five children were discussed in MDT in time for the optimal treatment window. Although the discussion section of this paper suggests possible treatment options for these children, there are significant technical barriers to decompression surgery and periorbital radiotherapy, and there are inconsistencies in their feasibility across different levels of care.

In summary, neuroblastoma with binocular blindness is rare in the clinic, mostly in children of young age, and is often associated with *MYCN* amplification and multiple metastases. Early hormone shock therapy and optic nerve decompression are beneficial for preserving the child's vision. A joint multidisciplinary consultation may help in the formulation of treatment decisions. Achieving a balance between good visual preservation and survival within the short optic nerve treatment window is extremely challenging.

## Data Availability

The dataset supporting the conclusions of this article is included within the article (Tables 1, 2). More detailed data can be obtained by contacting the author of the communication.
